# Supplementary material for: The influence of captivity on cardiac structure and function across age, in rhesus macaques
Source: bioRxiv. 2025 Oct 21:2025.10.20.683517. Preprint. [Version 1] doi: 10.1101/2025.10.20.683517 (PMC12633217; doi:10.1101/2025.10.20.683517)
Supplement: Supplement 1 [file media-1.pdf]

Supplementary Table 1. ANOVA P values for main effects and pairwise comparisons of cardiac structure and function in male and female young and old free-ranging rhesus macaques (extension of Table 2 in manuscript)

**Parameter**	**Interaction**	**Main effect**		**Age Pairwise comparison**		**Sex Pairwise comparison**	
	Age*sex	Age	Sex	Young vs old male	Young vs old female	Young Male vs female	Old male vs female
	P value	P value	P value	P value	P value	P value	P value
LV mass (g)	0.363	0.291	**< 0.001**			**< 0.001**	**< 0.001**
LV mass/body mass (g/kg$^{1.0}$)	0.247	**< 0.001**	0.808	**0.001**	0.112		
Aortic annulus diameter (mm)	0.385	**0.004**	**< 0.001**	**0.007**	0.150	**< 0.001**	**< 0.001**
Aortic annulus diameter/body mass (mm/kg$^{0.25}$)	0.275	**< 0.001**	**< 0.001**	**< 0.001**	**0.005**	**0.022**	**< 0.001**
IVSDd (mm)	0.731	**< 0.001**	0.123	**0.002**	**0.033**		
IVSDd/body mass (mm/kg$^{0.25}$)	0.735	**< 0.001**	0.790	**0.002**	**0.011**		
LVIDd (mm)	0.129	0.062	**< 0.001**			**< 0.001**	**< 0.001**
LVIDd/body mass (mm/kg$^{0.25}$)	0.072	0.585	**0.016**			0.626	**0.006**
PWDd (mm)	0.582	0.486	**0.002**			**0.004**	**0.004**
PWDd/body mass (mm/kg$^{0.25}$)	0.716	0.113	0.452				
RWT	0.285	**0.001**	0.304	0.116	**0.003**		
Sphericity index	0.839	0.241	**0.006**			**0.050**	**0.046**
**LV Volumes**							
LV EDV biplane (ml)	0.801	**< 0.001**	**< 0.001**	**0.004**	**0.016**	**< 0.001**	**< 0.001**
LV EDV/body mass (ml/kg$^{1.0}$)	0.724	0.102	0.087				
LV ESV biplane (ml)	0.279	**< 0.001**	**< 0.001**	**< 0.001**	0.030	**< 0.001**	**0.001**
LV ESV/body mass (ml/kg$^{1.0}$)	0.849	**0.022**	0.217	0.072	0.142		
LV SV biplane (ml)	0.670	0.066	**< 0.001**			**0.001**	**0.001**
LV SV/body mass (ml/kg$^{1.0}$)	0.663	0.677	0.091				
**LV Diastolic Function**							
E/A	0.942	**< 0.001**	0.427	**< 0.001**	**< 0.001**		
Transmitral E (m/s)	0.207	**< 0.001**	**0.013**	**0.020**	**< 0.001**	**0.004**	0.401
Transmitral A (m/s)	0.354	**0.006**	**0.001**	**0.008**	0.182	**0.002**	0.120
Septal e’ (mm/s)	0.799	**< 0.001**	0.716	**< 0.001**	**< 0.001**		
Septal a’ (mm/s)	**0.025**	**0.010**	0.957	**< 0.001**	0.803	0.075	0.145
Septal IVRT (ms)	0.692	**0.006**	0.330	0.090	**0.028**		
Lateral e’ (mm/s)	0.540	**< 0.001**	0.314	**< 0.001**	**< 0.001**		
Lateral a’ (mm/s)	**0.021**	**< 0.001**	0.893	**< 0.001**	**0.031**	0.059	0.147
Lateral IVRT (ms)	0.105	0.300	0.967				
**LV systolic function**							
LV EF Biplane (%)	0.273	**0.032**	0.075	**0.019**	0.459		
Septal s’ (mm/s)	0.113	**0.019**	0.068	0.569	**0.006**		
Lateral s’ (mm/s)	0.098	0.944	0.255				
Septal IVCT (ms)	0.250	0.624	**0.010**			**0.004**	0.331
Lateral IVCT (ms)	0.914	0.270	**< 0.001**			**0.009**	**0.018**
LV longitudinal strain (%)	0.760	0.404	0.167				
LV longitudinal strain rate (%/s)	0.842	0.410	0.231				
**LA volume and mechanics**							
LA max volume (ml)	0.493	0.374	**0.016**			0.194	0.035

LA max volume/body mass (ml/kg)	0.885	0.057	0.986				
LA strain R (%)	**0.029**	0.069	0.026	0.778	**0.006**	**0.002**	0.973
LA strain CD (%)	0.100	**0.002**	0.305	0.272	**0.001**		
LA strain CT (%)	0.265	0.065	0.097				

Supplementary Table 2. ANCOVA comparing cardiac structure and diastolic function between male and female rhesus macaques living in captive and free-ranging environments, using age as a covariate.

	Captive			Free-ranging					Main effect:
			Sex difference in captivity			Sex difference in free-ranging	Male Captive vs free-ranging	Female Captive vs free-ranging	i) Age*Env*Sex interaction<br>ii) Env*Sex interaction<br>iii) Environment<br>iv) Sex
	Male	Female	(P value)	Male	Female	(P value)	(P value)	(P value)	(P value)
**RWT**									i) **< 0.001**
Mean ± SD	0.49 ± 0.11	0.51 ± 0.10		0.33 ± 0.06	0.35 ± 0.07		**< 0.001**	**< 0.001**	ii) 0.952
adj. Mean ± SE	0.49 ± 0.01	0.51 ± 0.01		0.33 ± 0.01	0.34 ± 0.01				iii) **< 0.001**
									iv) 0.155
**IVSd (mm)**									i) **< 0.001**
Mean ± SD	5.8 ± 0.9	5.1 ± 1.0	**< 0.001**	3.9 ± 0.8	3.7 ± 0.8	0.264	**< 0.001**	**< 0.001**	**ii) 0.002**
adj. Mean ± SE	5.9 ± 0.1	5.1 ± 0.1		3.8 ± 0.1	3.7 ± 0.1				iii) **< 0.001**
									iv) <**0.001**
**LVIDd (mm)**									i) **< 0.001**
Mean ± SD	25.9 ± 4.2	21.9 ± 2.9	**< 0.001**	24.2 ± 2.0	22.4 ± 2.4	**< 0.001**	**0.004**	0.106	ii) < **0.001**
adj. Mean ± SE	25.7 ± 0.3	21.9 ± 0.2		24.3 ± 0.4	22.6 ± 0.4				iii) 0.256
									iv) **< 0.001**
**LVPWd (mm)**									i) **< 0.001**
Mean ± SD	6.4 ± 0.7	5.8 ± 0.9	**< 0.001**	4.1 ± 0.6	4.0 ± 0.7	**0.002**	**< 0.001**	**< 0.001**	ii) **0.009**
adj. Mean ± SE	6.4 ± 0.08	5.8 ± 0.05		4.1 ± 0.1	3.9 ± 0.1				iii) **< 0.001**
									iv) **< 0.001**
**E/A**									i) **< 0.001**
Mean ± SD	1.30 ± 0.37	1.15 ± 0.33	**0.020**	1.71 ± 0.48	1.65 ± 0.52	0.558	**< 0.001**	**< 0.001**	ii) 0.355
adj. Mean ± SE	1.23 ± 0.04	1.13 ± 0.02		1.75 ± 0.04	1.71 ± 0.04				iii) **< 0.001**
									iv) 0.063
**E (mm/s)**									i) **< 0.001**
Mean ± SD	0.74 ± 0.19	0.72 ± 0.16		0.68 ± 0.15	0.72 ± 0.16				ii) 0.183
adj. Mean ± SE	0.72 ± 0.02	0.71 ± 0.01		0.69 ± 0.02	0.73 ± 0.02				iii) 0.689
									iv) 0.414
**A (mm/s)**									i) **< 0.001**
Mean ± SD	0.58 ± 0.14	0.64 ± 0.17	**0.002**	0.42 ± 0.11	0.46 ± 0.12	0.208	**< 0.001**	**< 0.001**	ii) 0.385
adj. Mean ± SE	0.59 ± 0.02	0.65 ± 0.01		0.42 ± 0.02	0.48 ± 0.02				iii) **< 0.001**
									iv) **0.004**
**Avg e’ (mm/s)**									i) **< 0.001**
Mean ± SD	79 ± 21	79 ± 19		93 ± 18	91 ± 18		**< 0.001**	**< 0.001**	ii) 0.579
adj. Mean ± SE	77 ± 2	79 ± 1		94 ± 2	93 ± 2				iii) **< 0.001**
									iv) 0.966
**Avg a’ (mm/s)**									i) **< 0.001**
Mean ± SD	65 ± 14	70 ± 17		56 ± 15	55 ± 12		**< 0.001**	**< 0.001**	ii) 0.063
adj. Mean ± SE	66 ± 2	70 ± 1		56 ± 2	54 ± 2				iii) **< 0.001**
									iv) 0.376

RWT, relative wall thickness; IVSd, interventricular septal diameter in diastole; LVIDd, left ventricular internal diameter in diastole; LVPWd, left ventricular posterior wall thickness in diastole; E/A, ratio of early to later left ventricular filling velocity; E, early left ventricular filling velocity; A, late left ventricular filling velocity; Avg e’, an average of myocardial tissue velocity during early left ventricular filling taken at the septal and lateral wall; Avg a’, an average of myocardial tissue velocity during late left ventricular filling taken at the septal and lateral wall. Age covariate appearing in the model at age = 13.7

Supplementary Table 3. Slopes and intercepts of the relationship between cardiac variables and age in captive and free-ranging male and female rhesus macaques.

	Captive		Free-ranging	
**Parameter**	**Male**	**Female**	**Male**	**Female**
**RWT**				
Slope	0.011	0.008	0.005	0.006
Intercept	0.358	0.395	0.225	0.246
P value	**< 0.001**	**< 0.001**	**0.012**	**0.004**
**IVSd**				
Slope	0.034	0.052	0.073	0.047
Intercept	5.47	4.48	2.91	2.88
P value	0.071	**< 0.001**	**0.005**	**0.035**
**LVIDd**				
Slope	-0.244	-0.148	-0.118	-0.139
Intercept	29.0	24.1	26.1	24.2
P value	**0.004**	**< 0.001**	0.055	**0.009**
**LVPWd**				
Slope	0.058	0.037	0.003	0.037
Intercept	5.67	5.36	4.26	3.33
P value	**< 0.001**	**< 0.001**	0.896	0.119
**E/A**				
Slope	-0.033	-0.037	-0.084	-0.083
Intercept	1.71	1.64	2.96	2.87
P value	**< 0.001**	**< 0.001**	**< 0.001**	**< 0.001**
**E**				
Slope	-0.013	-0.010	-0.015	-0.021
Intercept	0.897	0.850	0.904	1.06
P value	**< 0.001**	**< 0.001**	**< 0.001**	**< 0.001**
**A**				
Slope	0.005	0.009	0.013	0.012
Intercept	0.513	0.522	0.226	0.315
P value	**0.045**	**< 0.001**	**< 0.001**	**0.002**
**Avg e’**				
Slope	-2.44	-1.82	-2.44	-2.82
Intercept	29.0	101	132	132
P value	**< 0.001**	**< 0.001**	**< 0.001**	**< 0.001**
**Avg a’**				
Slope	2.14	1.66	2.14	1.14
Intercept	23.5	48.4	23.5	39.3
P value	**< 0.001**	**< 0.001**	**< 0.001**	**0.004**
**Body mass**				
Slope	0.079	0.056	-0.018	-0.079
Intercept	12.4	8.59	10.5	8.37
P value	0.194	0.055	0.446	**0.004**

Supplementary Table 4. Comparison of slopes and intercepts of the relationship between cardiac variables and age between male and female captive and free-ranging rhesus macaques.

	**Captive**<br><br>**Male vs female**	**Free ranging**<br><br>**Male vs female**	**Males**<br><br>**Free-ranging vs captive**	**Females**<br><br>**Free-ranging vs captive**
**RWT**				
**Slopes**	P = 0.373	P = 0.732	**P < 0.001**	P = 0.521
**Intercepts**	P = 0.349	P = 0.862		**P < 0.001**
**IVSd**				
**Slopes**	P = 0.446	P = 0.455	P = 0.232	P = 0.960
**Intercepts**	**P < 0.001**	**P = 0.002**	P < **0.001**	P < **0.001**
**LVIDd**				
**Slopes**	P = 0.188	P = 0.793	P = 0.287	P = 0.892
**Intercepts**	**P < 0.001**	P = < **0.001**	P = **0.037**	P = 0.201
**LVPWd**				
**Slopes**	P = 0.294	P = 0.245	P = 0.306	P = 0.837
**Intercepts**	**P < 0.001**	**P < 0.001**	**P < 0.001**	**P < 0.001**
**E/A**				
**Slopes**	P = 0.818	P = 0.963	P < **0.001**	P < **0.001**
**Intercepts**	P = **0.001**	P = 0.236		
**E**				
**Slopes**	P = 0.479	P = 0.425	P = 0.667	P = 0.056
**Intercepts**	P = 0.641	P = **0.002**	P = 0.279	P = **0.028**
**A**				
**Slopes**	P = 0.317	P = 0.792	P = 0.088	P = 0.672
**Intercepts**	P = **0.004**	P = **0.001**	**P < 0.001**	**P < 0.001**
**e’**				
**Slopes**	P = 0.817	P = 0.526	P = 0.175	P = 0.094
**Intercepts**	P = 0.778	P = **0.014**	**P < 0.001**	**P < 0.001**
**a’**				
**Slopes**	P = 0.519	P = **0.041**	P = 0.074	P = 0.370
**Intercepts**	**P = 0.002**		**P < 0.001**	**P < 0.001**
